# Supplementary material for: Additive Modulated Perovskite Microstructures for High Performance Photodetectors
Source: Micromachines (Basel). 2020 Dec 10;11(12):1090. doi: 10.3390/mi11121090 (PMC7763584; doi:10.3390/mi11121090)
Supplement: Supplementary file 1 [file micromachines-11-01090-s001.pdf]

Supporting Information

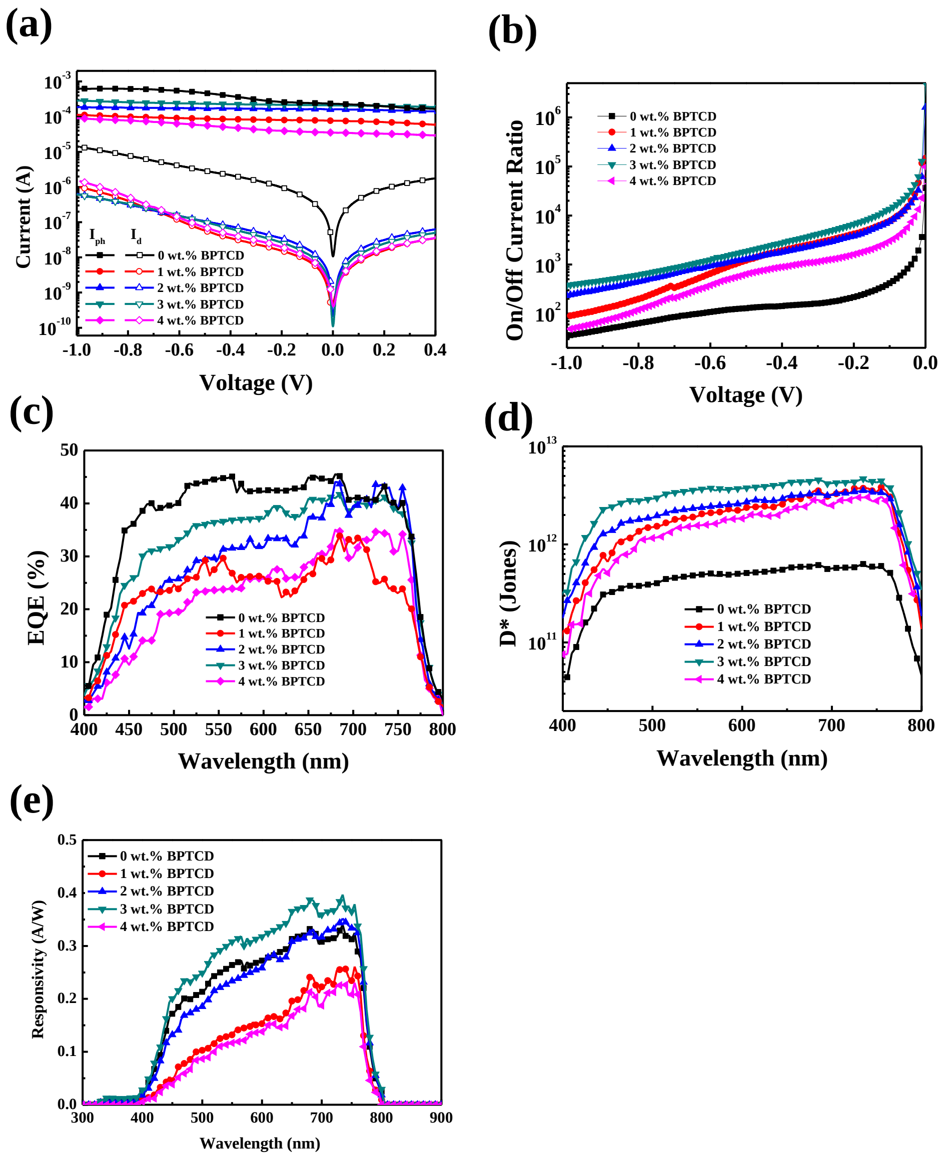

Figure S1. (a) Semilog J-V characteristics，(b) On/off current radio (c) Measured EQE spectra and (d) Calculated D* values at -0.1V of PePDs with various concentrations of BPTCD additive (e) Calculated responsivity values -0.1V of PePDs with various concentrations of BPTCD additive.

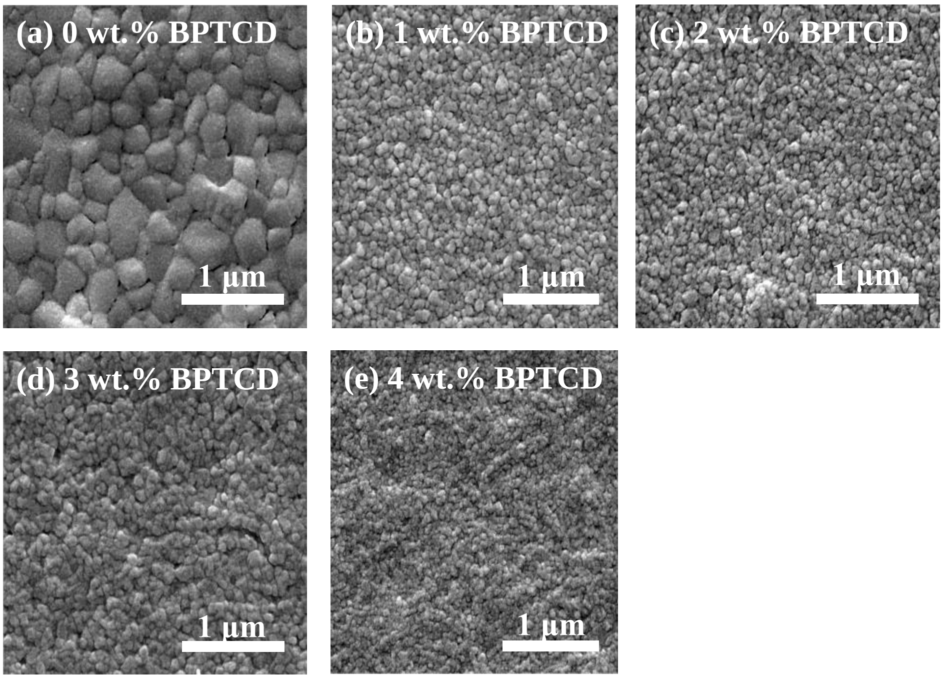

Figure S2. SEM images of perovskite film with different concentrations of BPTCD additive.

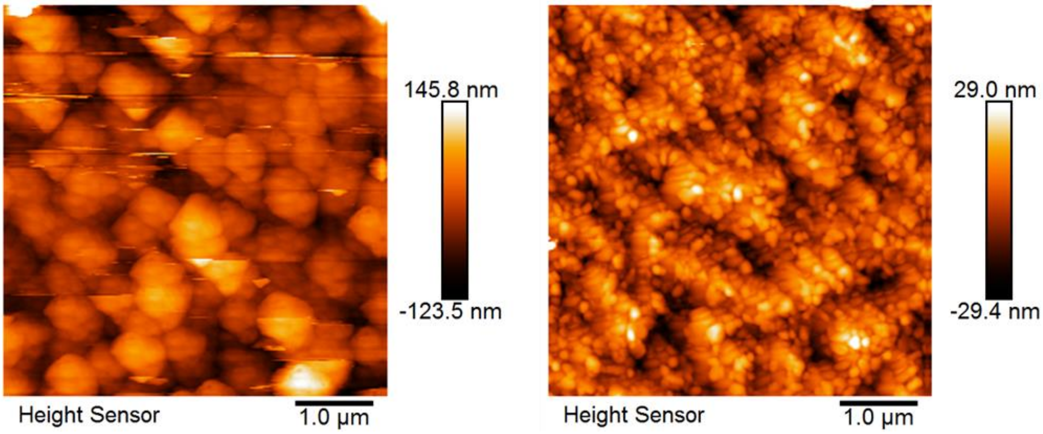

Figure S3. AFM measurement of perovskite films with 3% BPTCD and without BPTCD.
